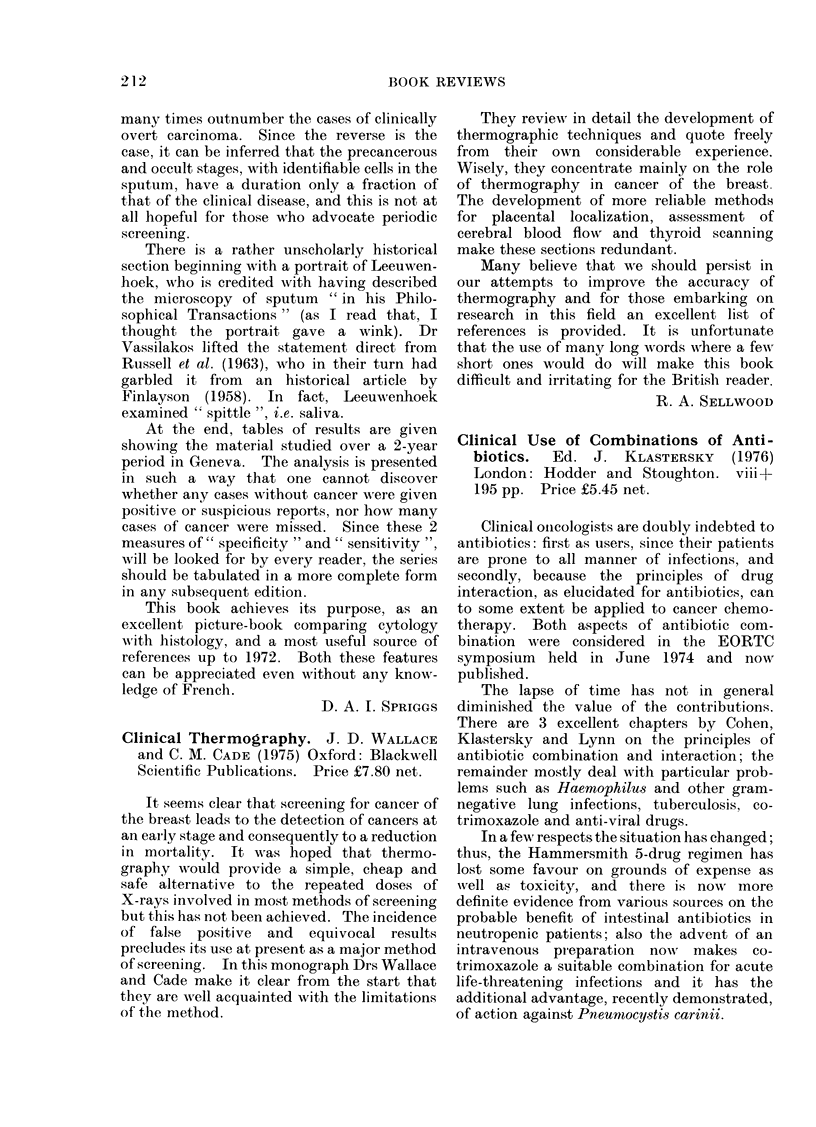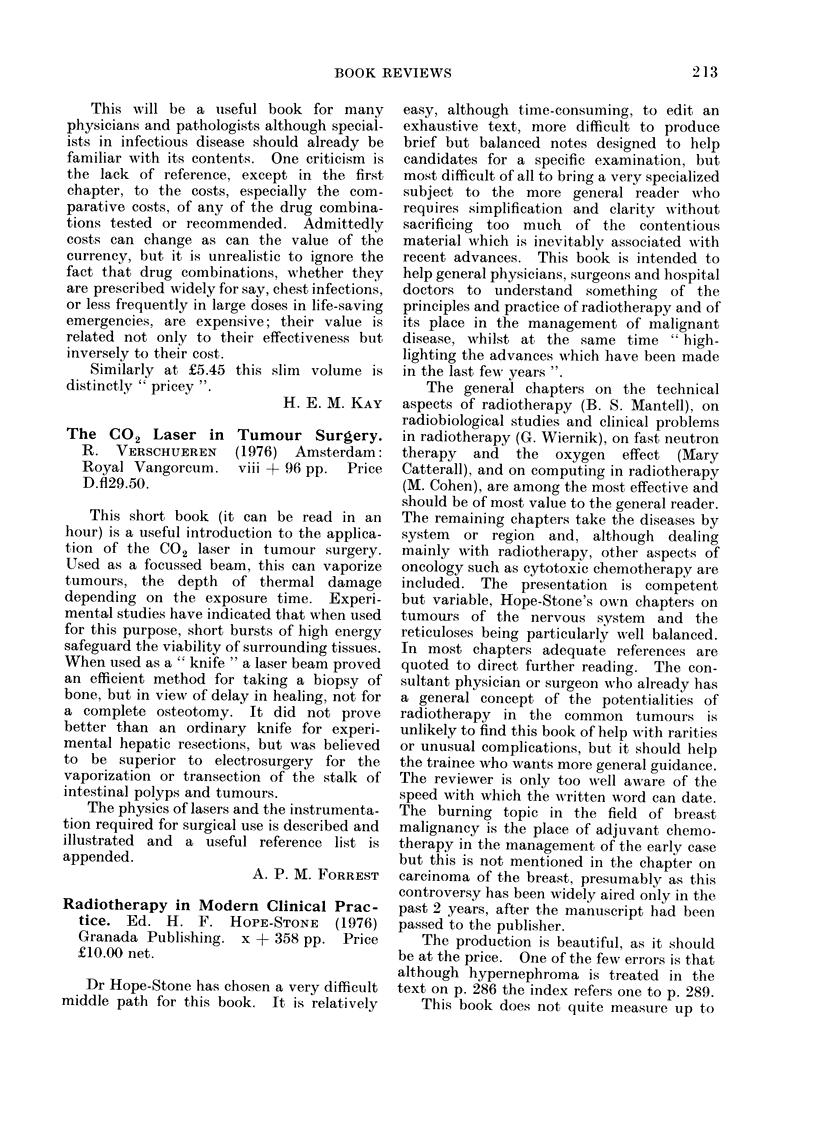# Clinical Use of Combinations of Antibiotics

**Published:** 1976-08

**Authors:** H. E. M. Kay


**Clinical Use of Combinations of Antibiotics.** Ed. J. KLASTERSKY (1976) London: Hodder and Stoughton. viii+195 pp. Price £5.45 net.

Clinical oncologists are doubly indebted to antibiotics: first as users, since their patients are prone to all manner of infections, and secondly, because the principles of drug interaction, as elucidated for antibiotics, can to some extent be applied to cancer chemotherapy. Both aspects of antibiotic combination were considered in the EORTC symposium held in June 1974 and now published.

The lapse of time has not in general diminished the value of the contributions. There are 3 excellent chapters by Cohen, Klastersky and Lynn on the principles of antibiotic combination and interaction; the remainder mostly deal with particular problems such as *Haemophilus* and other gram-negative lung infections, tuberculosis, co-trimoxazole and anti-viral drugs.

In a few respects the situation has changed; thus, the Hammersmith 5-drug regimen has lost some favour on grounds of expense as well as toxicity, and there is now more definite evidence from various sources on the probable benefit of intestinal antibiotics in neutropenic patients; also the advent of an intravenous preparation now makes co-trimoxazole a suitable combination for acute life-threatening infections and it has the additional advantage, recently demonstrated, of action against *Pneumocystis carinii.*

This will be a useful book for many physicians and pathologists although specialists in infectious disease should already be familiar with its contents. One criticism is the lack of reference, except in the first chapter, to the costs, especially the comparative costs, of any of the drug combinations tested or recommended. Admittedly costs can change as can the value of the currency, but it is unrealistic to ignore the fact that drug combinations, whether they are prescribed widely for say, chest infections, or less frequently in large doses in life-saving emergencies, are expensive; their value is related not only to their effectiveness but inversely to their cost.

Similarly at £5.45 this slim volume is distinctly " pricey ".

H. E. M. KAY